# Evaluation of physical, chemical, and color‐matching properties of monochromatic resin composites: An in vitro study

**DOI:** 10.1111/eos.70081

**Published:** 2026-03-08

**Authors:** Ericka dos Santos Lopes, Mayara Evelly Duarte de Lima, Emanuel Ewerton Mendonça Vasconcelos, Natália Gomes de Oliveira, Gabriela Queiroz de Melo Monteiro, Luís Felipe Espíndola‐Castro

**Affiliations:** ^1^ School of Dentistry Universidade Federal de Pernambuco Recife Brazil; ^2^ School of Dentistry Universidade de Pernambuco Recife Brazil

**Keywords:** color stability, microhardness, resin composites, solubility, sorption

## Abstract

The objectives of this study are to evaluate, in vitro, the microhardness, sorption, solubility, color stability, and color‐matching ability of monochromatic resin composites: Palfique Omnichroma / Tokuyama (Mono1) and Vittra APS Unique / FGM (Mono2) compared with the conventional resin composite: Filtek Z250 XT / Solventum (Conv). For this analysis, ten disc‐shaped composite specimens were prepared for each group for the microhardness, sorption, solubility, and color stability tests. To evaluate color‐matching ability, 36 tooth specimens were obtained from the buccal/lingual surfaces of human molars (*n* = 12). The teeth were restored, and color compatibility was assessed with a digital spectrophotometer (Vita Easy Shade) and visually. Microhardness and sorption were higher in the Conv group. However, sorption values are within the limits established by ISO 4049/2019 for all groups. No significant difference in solubility or visual color match was noted. However, the conv group demonstrated superior color‐matching in the spectrophotometer analysis. After 7 days of coffee immersion, the Mono 1 group showed slightly less pigmentation. Monochromatic resin composites demonstrated physicochemical and organoleptic properties comparable to those of the evaluated conventional resin composites, suggesting similar compatibility and a potential option for simplifying clinical restorative protocols.

## INTRODUCTION

The composition of resin composites has evolved substantially since their introduction into dentistry more than six decades ago [1, 2]. The polychromatic nature of natural teeth requires restorative systems that include multiple shades, commonly referenced using the VITA Classic shade guide [3]. However, accurate shade selection of restorative materials remains challenging for clinicians.

Furthermore, resin composites are available in different opacities, generally classified as dentin (opaque), enamel (translucent), or body (intermediate opacity and translucency), in order to mimic the optical properties of a natural tooth [4, 5]. This variety of shades complicates the color‐matching process and increases chairside time [6]. Additionally, determining the appropriate shades depends on several factors, such as adequate lighting, the distance between the observer and the substrate, and clinicians’ experience, visual acuity, fatigue, and mood [7].

From this perspective, in an effort to shorten the restorative procedure time, simplify color matching, and reduce costs and resin storage, monochromatic resin composites (single‐shade materials) capable of covering a wide range of classic shades of the VITA scale, from A1 to D4, have been developed [8]. Resin composites that adapt their color to match that of adjacent hard dental tissues may have a clinical advantage, as they can improve the esthetic appearance of restorations, simplify color matching by reducing errors associated with shade selection, and mitigate shade mismatches [9, 10].

Monochromatic resins are designed to allow the restorative material naturally mimic a wide range of tooth colors within the VITA scale through light reflection [10]. This structural color phenomenon results from wavelength discrimination arising from interactions between incident light and nanostructures, such as thin films, diffraction gratings, or photonic crystals [11]. For example, a monochromatic resin composite contains uniformly sized supra‐nanosphere filler particles of silicon dioxide (SiO_2_) and zirconia (ZrO_2_) with a particle size of approximately 260 nm. These particles interact with incident light and modify its transmission within the red‐to‐yellow region of the color spectrum, allowing the material to blend with the colors of the surrounding tooth structures [8].

Although these materials facilitate color matching, their physical, chemical, and optical properties must also be appropriate. Regardless of resin type, all are subject to degradation due to the complex nature of the oral environment, where saliva, foods and beverages, temperature fluctuations, and masticatory loads can adversely affect clinical performance [12]. Discoloration may arise from intrinsic factors associated with the resin material and inadequate photopolymerization, as well as extrinsic factors associated with dietary habits [13].

Degradation processes involve the composition of the materials, such as resin matrix, filler, and bonding agent. Extrinsic factors relate to color change resulting from the pigmentation of certain foods and beverages, such as curry, açaí, red wine, and coffee [10]. Material aging typically manifests as the leaching of degradation products and unreacted substrates, reduced strength and hardness, increased roughness and abrasiveness, color changes, increased water sorption, and cracking [12]. Clinically, resin degradation, particularly color changes and loss of luster, is one of the main reasons for the replacement of restorations in anterior teeth [14].

Sorption and solubility can be considered precursors of several chemical and physical processes that damage the polymeric structure, potentially compromising its clinical effectiveness and longevity. These phenomena are influenced by insufficient monomer conversion during photopolymerization and inadequate depth of cure [13].

Therefore, this in vitro study aimed to evaluate the microhardness, sorption, solubility, color stability, and color‐matching ability of two monochromatic resin composites, in comparison with the conventional resin composite. The null hypothesis tested was that there would be no statistically significant differences (*p* < 0.005) among the evaluated resin composites in terms of (I) microhardness, (II) sorption, (III) solubility, (IV) color stability, and (V) color‐matching ability.

## MATERIALS AND METHODS

The study was conducted in accordance with the ethical guidelines established by Resolution 466/12 of the National Health Council and was approved by the Human Research Ethics Committee of the Federal University of Pernambuco under opinion no. 4.717.537.

Two commercially available monochromatic resin composites (Mono1: Palfique Omnichroma, Tokuyama; and Mono2: Vittra APS Unique, FGM) and one conventional resin composite (Conv: Filtek Z250XT, Solventum) were used for the experiments (Table 1).

**TABLE 1:** Materials used in the study according to the manufacturer's instructions.

Resin composites (Group)	Color System	Organic matrix	Inorganic filler	Filler % wt/vol	Manufacturer batch number
Palfique Omnichroma (Mono1)	Single Shade (Single resin reaches from A1to D4)	UDMA, TEGDMA, Mequinol, Dibutyl hydroxyl toluene, and UV absorber.	Spherical silica zirconia filler (mean particle size: 260 nm). Nanoparticle.	79%/68%	089E33
Vittra APS Unique (Mono2)	Single Shade (Single resin reaches from Bleach to D4)	UDMA, PEGDMA, TEGDMA, APS System, fluorescent agents, pigments, stabilizers. Initiators and silane.	Zirconia nanospheres, with an average particle size of 200 nm. Submicrohybrid	72%–80%/52%–60%	290,923
Filtek Z250 XT (Conv)	Multi Shade (Available colors: A1, B1, OA2, A2, C2, B2, B3, D3, OA3, A3, A3.5, A4)	BIS‐GMA, BIS‐EMA, UDMA, PEGMA, TEG‐DMA, fluorescent agents, pigments, stabilizers, and initiators.	Particle size range of 0.01 to 3.5 µm. Treated silanized ceramic, Treated sílica. Microhybrid.	81.8%/68%	2,412,200,357; 2,324,300,140

Abbreviations: APS, advanced polymerization system; Bis‐EMA, bisphenol hydroxyethyl methacrylate; Bis‐GMA, bisphenol A‐glycidyl methacrylate; PEGMA, polyethyleneglycol methyl ether methacrylate; TEGDMA, triethylene glycol dimethacrylate; TEGDMA, triethylene glycol dimethacrylate; UDMA, urethane‐dimethacrylate.

### Sample size calculation and specimen preparation

Regarding the evaluation of microhardness, color stability, sorption, and solubility, 10 specimens of each material (*n* = 10) were prepared. The sample size was determined based on the experimental design adopted in previous studies [13, 15, 16, 17] to optimize the use of the tested materials. As ISO 4049:2019 recommends a sample size of *n* = 5 for sorption and solubility tests, in which specimens are immersed in water, these same specimens were used as the control group for color stability analysis. Additionally, five extra specimens of each material were prepared for immersion in coffee; these specimens were not used in the sorption and solubility tests. Thus, prior to the sorption, solubility, and color stability tests, the microhardness of all ten specimens was evaluated.

Following the recommendations of ISO 4049:2019, the specimens were prepared using a bipartite metal mold (Ø = 15 mm × 1 mm) positioned between two polyester matrix strips and two glass plates (500 g). Photopolymerization was performed in the center of all specimens using a light‐emitting diode source (Gran Valo, Ultradent) at an irradiance of 1200 mW/cm^2^ for 40 s, on each side of the specimens. After removing the specimen from the metal mold, the excess material was removed using silicon carbide sandpaper with decreasing grit sizes (#600, 1000, and 1500). The final dimension of the specimen was confirmed with a digital caliper (± 0.01 mm; MDC‐25 M, Mitutoyo).

For the color‐matching test, the size of the tooth specimens was calculated based on the color variation results reported by Altınışık, Özyurt [18]. Using the website http://estatistica.bauru.usp.br/calculoamostral, a standard deviation of 0.4 (∆E) and a minimum difference to be detected of 0.6 were considered. An alpha value of 5% and a power of 80% illustrated the need for ten specimens per group. An additional 20% was added to consider possible losses, with 12 per group. For this purpose, 18 human molars were used. The roots of the teeth were removed and sectioned longitudinally to ensure that the vestibular and lingual surfaces of each tooth were separated, resulting in 36 tooth specimens that were randomly distributed into one of the three experimental groups (*n* = 12). The tooth specimens were fixed in PVC cylinders with acrylic resin, with the vestibular/lingual surface facing upward. Healthy human molars extracted for therapeutic reasons, such as orthodontic treatment, periodontal disease, or third molar erupted removal, were included in this analysis. Teeth presenting carious lesions, structural cracks, or requiring odontosection of the clinical crown during the surgical procedure were excluded.

### Vickers microhardness test

Vickers microhardness was measured on the resin composite surface using a digital microhardness tester (ISH‐MR 150/INSIZE). The indenter was positioned at the center of each specimen (*n* = 10), and a load of 300 gf was applied for 15 s. Three indentations 5 mm apart were made on each specimen. The mean of the three measurements was used as the specimen's microhardness value [13, 15, 16, 17].

### Sorption and solubility

**FIGURE 1:**
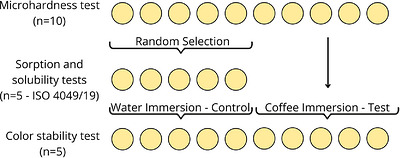
Flowchart of specimen allocation.

After the microhardness evaluations, five specimens were randomly selected for the sorption and solubility tests. The specimens were allocated according to the flowchart shown in Figure 1, which was developed based on previous studies [13, 15, 16, 17]. Randomization was performed using the website https://sites.usp.br/praticaempesquisa/tabela‐de‐numeros‐aleatorios/, where the specimens were numbered from 1 to 10, and five numbers were drawn to carry out the tests. According to ISO 4049:2019, the specimens were placed in a desiccator containing silica gel at 37°C ± 2°C. After 24 h, the specimens were weighed daily until they reached a constant mass on an analytical balance (+0.01 mg; AUW 220D, Shimadzu Analytical Balance) (m1), with variations of < 0.1 mg. This constant mass (m1) was taken as the initial specimen mass and expressed in micrograms (µg). The specimens were then stored in 50‐mL Falcon tubes filled with distilled water and kept at 37°C for 7 days. Afterward, they were removed, gently dried with filter paper, weighed again, and the mass was recorded (m2). The specimens were returned to the desiccator and weighed daily until reaching a constant mass (m3). Sorption (SP) and solubility (SL) (µg/mm^3^) were calculated using the following equations:

$$Sp=\left(m2-m1\right)/V\;and\;SL=\left(m1-m3\right)/V$$

Here, the volume of the specimens was calculated in mm^3^ (*V* = π*r* ^2^ *h*), where r is the average radius of the specimen (diameter/2) and *h* is the average thickness of the specimen.

### Color stability

The color stability analysis was based on previous studies [13, 15, 16, 17] using the CIELAB color space and a digital spectrophotometer (VITA Easy Shade; Wilcos). Color measurements were performed by a single operator (1) before immersion, (2) after 1 day, and (3) after 1 week of storage in distilled water and coffee (replaced daily, at room temperature). The five specimens (n = 5) used for sorption and solubility evaluations and immersed in distilled water served as the control group. The remaining five specimens evaluated for microhardness, not selected for sorption and solubility tests, were immersed in coffee. The specimens were placed on white photographic paper (Kodak, New York, USA), and three measurements were taken in the central area and averaged. The coffee was prepared by dissolving 0.51 g of instant coffee powder in 50 mL of distilled water (Nescafé, Nestlé). The CIELAB system is composed of three axes: L* (lightness from 0 = black to ´ 100 = white), a* (from *a* = green to ´ +*a* = red), and b* (from *b* = blue to ´ +*b* = yellow). The color change (∆E) was calculated using the following equation:

$$\Delta E={\left[{\left(L*1-L*2\right)}^{2}+{\left(a*1-a*2\right)}^{2}+{\left(b*1-b*2\right)}^{2}\right]}^{1/2}$$

L*2−L*1 = (final reading minus initial reading) a*2−a*1 = (final reading minus initial reading) b*2−b*1 = (final reading minus initial reading).

### Instrumental color‐matching evaluation

The vestibular/lingual tooth specimens had cavities prepared (5 mm in diameter and 2 mm in depth) using a diamond burr, type #3053 (KG Sorensen). The cavities were restored with composites following the manufacturer's instructions and light‐cured with a light‐emitting diode source (Gran Valo, Ultradent) with an irradiance of 1200 mW/cm^2^ for 40 s. For the monochromatic resin groups, no shade selection test was performed. For the Conv group, however, the resin shade was selected by inserting a small increment on the specimen surface.

The restored tooth surfaces were evaluated using the digital spectrophotometer VITA Easyshade before and after performing the restorative procedure (two assessments were performed at each time point, and averaged). The color was recorded following the same parameters described in the color stability analysis and calculation of color variations. Therefore, a lower ∆e indicates that the resin composite approached the initial color of the remaining tooth structure.

### Visual color‐matching assessment

**FIGURE 2:**
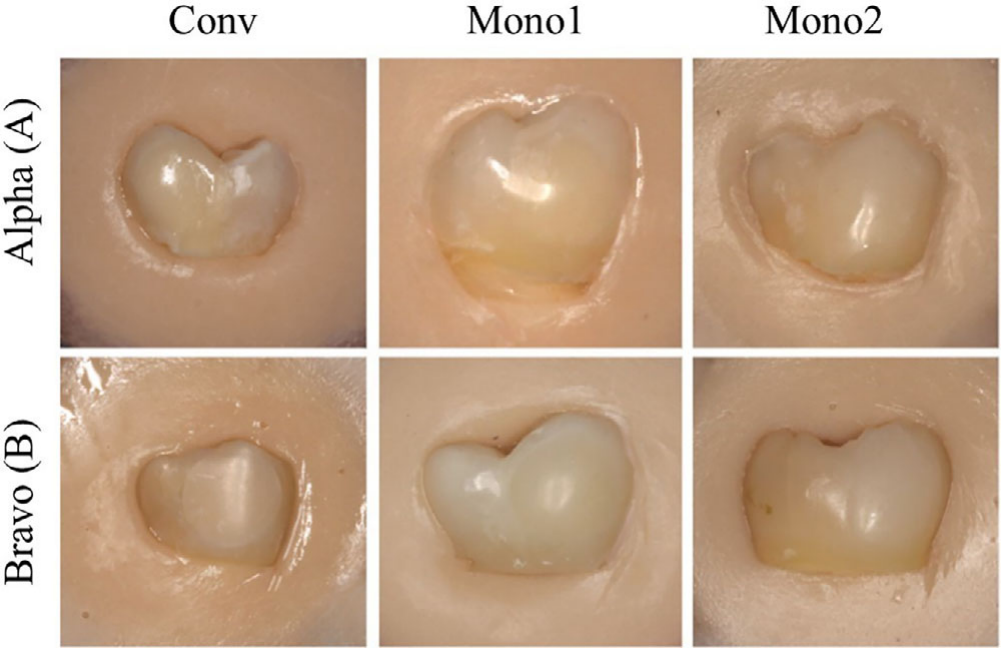
Color‐matching analysis based on USPHS criteria. Images of specimens from the three tested materials that received Alpha (A) and Bravo (B) ratings according to the criteria described in Table 2.

After the restorative procedure, the restored teeth were evaluated by two blinded evaluators. Before evaluation, examiners underwent training using a set of standardized images and participated in calibration exercises. Inter‐ and intra‐examiner agreement was assessed by calculating Cohen's kappa (κ = 0.81). The evaluators were blinded to the materials tested in this study, and the code on each specimen was covered with opaque adhesive tape. The specimens were positioned 30 cm from the evaluators, who observed the specimens for 3 s. Disagreements between the two were resolved by a third independent evaluator. The restorations were evaluated according to the criteria established by the United States Public Health Service (USPHS). The restorations were classified based on the following criteria: Alpha (A) for those considered ideal, Bravo (B) for those clinically acceptable, and Charlie (C) for those clinically unacceptable, as described in Table 2. The visual color‐matching criteria used in the visual analysis are shown in Figure 2.

**TABLE 2:** United States Public Health Service criteria for color matching.

Score	Parameters
Alpha (A)	The restoration harmonizes with the color and translucency of the adjacent dental tissues, ensuring an excellent esthetic result.
Bravo (B)	The restoration harmonizes acceptably with the color and translucency of the adjacent tooth tissues.
Charlie (C)	The restoration does not adequately harmonize with the color and translucency of the dental tissues, severely compromising the esthetic result.

### Statistical analysis

Data were analyzed descriptively using mean and standard deviation for quantitative variables and absolute and relative frequencies for categorical variables.

Normality of the data distribution was assessed using the Shapiro–Wilk test, and homogeneity of variances was evaluated using Levene's test. The choice of statistical tests was based on data distribution and the number of comparison groups.

For microhardness, which showed normal distribution and homogeneity of variances, comparisons among materials were performed using one‐way analysis of variance (ANOVA), followed by Tukey's post hoc test for multiple comparisons.

For sorption and solubility, as the assumptions of normality were not met, comparisons among materials were performed using the Kruskal–Wallis test, followed by Conover's post hoc test when significant differences were detected.

For color stability analysis (ΔE), data were analyzed considering the effects of material, solution (water or coffee), and evaluation time (1 and 7 days). Comparisons between evaluation times within the same group and solution were performed using the paired *t*‐test. Comparisons between solutions (water vs. coffee) within each group and evaluation time were conducted using the *t*‐test assuming equal variances. For comparisons among groups at each evaluation time, different statistical approaches were applied according to the solution. In the water solution, group comparisons were performed using one‐way analysis of variance (ANOVA), followed by Tukey's post hoc test. In the coffee solution, group comparisons were carried out using the Kruskal–Wallis test, followed by Conover's post hoc test.

For the instrumental color‐matching evaluation, the *F*‐test (one‐way analysis of variance—ANOVA) was used to compare the mean values among the experimental groups. When statistically significant differences were detected, multiple comparisons were performed using Tukey's post hoc test. And to visualize color‐matching (categorical data) was analyzed using Fisher's exact test.

All statistical tests were performed with a significance level set at *α* = 0.05. Statistical analyses were conducted using IBM SPSS Statistics version 27 (IBM Corp., Armonk, NY, USA) and MedCalc version 20.104 (MedCalc Software, Ostend, Belgium).

## RESULTS

The results of the microhardness, sorption, and solubility tests of the tested materials are presented in Table 3. Higher microhardness values were observed in the Conv group (average 80.5) than in Mono2 (average 57.1) and Mono1 (average 48.8). This difference was significant between the resins (*p* < 0.001). The sorption values were significantly higher in the Conv group (21.40 µg/mm^3^) (*p* = 0.007) than in Mono1 (15.3 µg/mm^3^) and Mono2 (15.5 µg/mm^3^). Solubility results were also higher in the Conv group (average 1.6 µg/mm^3^) than in Mono1 (1.1 µg/mm^3^) and Mono2 (1.1 µg/mm^3^); however, the differences were not significant.

**TABLE 3:** Vickers microhardness and sorption and solubility analysis (µg/mm^3^).

Group	Microhardness Mean ± SD	Sorption Mean ± SD	Solubility Mean ± SD
Conv	80.5 ± 2.1 ^(A)^	21.4 ± 0.6 ^(A)^	1.6 ± 0.7 ^(A)^
Mono1	48.8 ± 1.8 ^(B)^	15.5 ± 0.5 ^(B)^	1.1 ± 0.8 ^(A)^
Mono2	57.1 ± 1.6 ^(C)^	15.3 ± 1.3 ^(B)^	1.1 ± 1.1 ^(A)^
***p*‐value**	***p*** ^a^ ** < 0.001** ^*^	***p*** ^b^ ** = 0.007** ^*^	***p*** ^b^ ** = 0.638**

*Note*: Distinct superscript capital letters mean statistically significant differences.

^a^ *F*‐test (ANOVA) with Tukey comparisons.

^b^ Kruskal–Wallis test with Conover test comparisons.

* Significant difference at 5%.

Table 4 presents the results of the color stability tests, calculated through color variations (ΔE) after 1 and 7 days of immersion in distilled water (control) and coffee. The average color variation over 1 day (ΔE1) in each material was substantially higher when the specimens were immersed in coffee. These results indicate significant differences between the solutions for each resin composite (*p* < 0.001). The difference between the resins in the coffee solution was not significant (*p* = 0.071).

**TABLE 4:** Color stability analysis (ΔE) at 1 day (ΔE1) and 7 days (ΔE7).

Group	Solution	ΔE1	ΔE7	*p*‐value
Conv	Water	2.6 ± 0.3 ^(A)^	3.0 ± 0.4 ^(A)^	***p*** ^a^ ** = 0.035** ^*^
	Coffee	9.3 ± 0.6 ^(a)^	13.7 ± 1.8 ^(a)^	***p*** ^a^ ** = 0.004** ^*^
***p*‐value**		***p*** ^b^ ** < 0,001** ^*^	***p*** ^b^ ** < 0,001** ^*^	
Mono1	Water	0.4 ± 0.2 ^(B)^	2.6 ± 0.2 ^(A)^	***p*** ^a^ ** < 0.001** ^*^
	Coffee	9.3 ± 0.4 ^(a)^	11.6 ± 0.5 ^(b)^	***p*** ^a^ ** = 0.002** ^*^
***p*‐value**		***p*** ^b^ ** < 0,001** ^*^	***p*** ^b^ ** < 0,001** ^*^	
Mono 2	Water	1.0 ± 0.3 ^(C)^	0.5 ± 0.2 ^(B)^	***p*** ^a^ ** = 0.009** ^*^
	Coffee	10.1 ± 0.6 ^(a)^	13.7 ± 1.1 ^(a)^	***p*** ^a^ ** = 0.002** ^*^
***p*‐value**		***p*** ^b^ ** < 0,001** ^*^	***p*** ^b^ ** < 0,001** ^*^	
***p*‐value**		***p*** ^c^ ** < 0.002** ^*^	***p*** ^c^ ** < 0.004** ^*^	
***p*‐value**		***p*** ^d^ ** = 0.071**	***p*** ^d^ ** = 0.038** ^*^	

*Note*: Different uppercase letters in parentheses indicate significant differences among groups in the water solution, whereas different lowercase letters indicate significant differences among groups in the coffee solution.

^a^ Paired *t*‐test for comparisons between evaluation times within each group and solution.

^b^ *t*‐Test assuming equal variances for comparisons between solutions within each group and evaluation time.

^c^ One‐way ANOVA (F test) for comparisons among groups at each evaluation time in the water solution, followed by Tukey's post hoc test.

^d^ Kruskal–Wallis test for comparisons among groups at each evaluation time in the coffee solution, followed by Conover's post hoc test.

* Significant difference at the 5% level.

In the 7‐day analysis, color variability in each material after 7 days (ΔE7) was much higher when immersed in coffee than in distilled water. These results indicate significant differences between the solutions for each resin composite (*p* < 0.001). Mono1 showed slightly less pigmentation after 7 days of immersion in coffee (*p* = 0.038).

In the comparative analysis performed using the Paired *t*‐test, considering the 1‐ and 7‐day evaluations, all materials showed statistically significant differences, regardless of the immersion solution.

Table 5 presents the results of the color‐matching analysis of the tested resins according to different analysis systems (instrumental and visual). In the instrumental analysis, Conv group showed lower ∆E values, indicating greater color reproduction capacity (5.3), whereas the mean values were 13.9 and 15.4 for Mono2 and Mono1, respectively (*p* < 0.001).

**TABLE 5:** Color‐matching in the instrumental (digital spectrophotometer) and visual analyses.

		Visual analysis *N* (%)		
Group	Instrumental analysis Mean ± SD	A (Alfa)	B (Bravo)	C (Charlie)
Conv	5.3 ± 2.15 ^(A)^	9 (75)	3 (25)	‐ (0)
Mono1	15.4 ± 2.60 ^(B)^	8 (66.7)	4 (33.3)	‐ (0)
Mono2	13.9 ± 3.10 ^(B)^	8 (66.7)	4 (33.3)	‐ (0)
***p*‐value**	***p*** ^a^ ** < 0.001** ^*^	***p*** ^b^ ** = 1.000**		

*Note*: If the superscript capital letters in parentheses are distinct, significant differences between the materials are proven.

^a^ *F*‐test (one‐way ANOVA) followed by Tukey's multiple comparison test.

^b^ Fisher's Exact Test.

* Significant difference at 5%.

In the visual color analysis, the majority of specimens in each group were classified in the alpha category, with values ranging from 66.7% to 75.0%, whereas the remainder were assigned to the bravo category. No specimen was classified in the Charlie category. Furthermore, no significant differences (*p* = 1.0) were observed between the resins analyzed.

## DISCUSSION

The first null hypothesis was rejected, as the Conv group (Filtek Z250 XT resin composite) exhibited significantly higher microhardness values compared with the other tested materials (*p* < 0.001). The composition of resins significantly influences their physical and mechanical characteristics. Filler particles are added to decrease water sorption, reduce abrasion, and improve microhardness [19]. The performance of the restorative material is determined by characteristics such as size, distribution, and volume of the filler [20]. As shown in Table 1, the Filtek Z250 contains a greater proportion of filler particles, which may explain the higher microhardness values in this group.

Furthermore, this resin (Z250) has silane‐treated zirconia filler particles in microhybrid size. An in vitro study comparing the microhardness of microhybrid and nanoparticulate resins immersed in different solutions revealed that the microhybrid resin exhibited higher resistance values [21]. Furthermore, zirconia is known for its high mechanical strength, and its incorporation into resin composites can increase their microhardness [22].

The second null hypothesis was also rejected, as the Conv group exhibited significantly higher sorption values compared with the tested monochromatic resin composites (*p* = 0.007). Sorption is important for the clinical success of dental materials. Although resin composites are considered stable and impermeable, water sorption occurs after the curing process because of the hydrophilic nature of the organic matrix, which induces water absorption and swelling in the spaces between polymer chains. Al‐Odayni et al. indicated that composites made with BisGMA and BisEMA monomers exhibit high viscosity and hydrophilicity, which can trigger chemical degradation by breaking the resin‐filler bonds, resulting in the erosion of the organic matrix and the release of unreacted monomers into the oral environment [23].

This phenomenon may justify the results of the present study because Z250 resin composite demonstrated greater sorption than other resins and is composed of large quantities of hydrophilic monomers, which are absent in the composition of the other materials (Table 1). A study attributed the lower sorption of the other resins to the presence of hydrophobic monomers in their compositions, such as UDMA [24]. Despite presenting greater sorption than monochromatic resins, Z250 resin composite did not exceed the limit established by ISO 4049/2019, which recommends sorption values not exceeding 40 µg/mm^3^.

The third null hypothesis was accepted. Regarding solubility (Table 3), no difference was found between the groups, and this finding suggests that monochromatic resins have a solubility level close to that of a conventional resin. Although they appear to be directly proportional, materials with high sorption do not necessarily demonstrate high solubility and vice versa [25]. Solubility is related to the degree of polymer conversion; molecular size, type, and amount of the inorganic matrix; monomeric hydrophilicity; and leaching of unpolymerized monomers [26]. A study that tested the solubility of 11 resins identified that Z250 met the solubility limits established by ISO 4049/2019 (7.5 µg/mm^3^), corroborating the results of the present study [24].

Resin composites with smaller filler particles are expected to be less susceptible to staining. This is because the even distribution of smaller particle sizes within the resin matrix reduces the exposure of this matrix to the oral environment, which favors low sorption and, consequently, lower incorporation of pigments [27]. The color stability test was performed with a spectrophotometer, a sensitive method that measures color accurately and is based on the measurement of the spectral reflectance or transmittance of a specimen [28].

The fourth null hypothesis was partially accepted. As presented in Table 4, no difference was found between the groups after 1 day of immersion in coffee. However, the Mono 1 group exhibited lower pigmentation after 7 days of evaluation following immersion in coffee. These findings may be related to material sorption, as the Mono 1 group exhibited significantly lower sorption than the conventional group (Table 3). Moghaddasi et al. indicated that the greater the sorption, the greater the susceptibility to staining [29]. Sorption is determined by the ability to absorb liquids into the resin matrix and, with it, the pigments.

According to Souza et al., when the color variation value is > 3.3 (∆E > 3.3), the change is detectable and considered clinically unacceptable [17]. Therefore, after 7 days, all specimens presented values above this threshold when immersed in coffee. However, to consider restoration as unacceptable in terms of color, other factors must be taken into account, such as the type of tooth and cavity restored and the patient's expectations. Just as resin composite pigments are immersed in coffee, a tooth also darkens because of enamel permeability. Therefore, resin staining can follow the natural darkening of the teeth and should not be considered unacceptable.

These findings demonstrated that all tested materials showed statistically significant differences in immersion time, regardless of the solution tested (water or coffee). However, it is important to emphasize that, although statistically significant differences were observed, specimens immersed in water for 1 and 7 days exhibited color changes below the clinically perceptible threshold (ΔE < 3.3).

Our results regarding monochromatic resins are consistent with those of Santana et al., who concluded that Mono1 and Mono2 resins exhibited similar color stability [30]. In a randomized clinical study, Anwar et al. analyzed Mono1 resin over 12 months and found a gradual reduction in its color stability; however, the change was within clinically acceptable parameters [31].

The fifth null hypothesis was partially accepted. In the analysis of the ability of resin composites to reproduce the color of adjacent dental tissues (Table 5) in the instrumental analysis (digital spectrophotometer), Z250 presented a better potential than monochromatic resins. However, in the visual analysis, no significant differences were observed among the materials tested, with all resins classified as alpha and bravo (clinically acceptable). These findings are consistent with those reported by Santana et al., who found that no Charlie ratings were assigned to the monochromatic resin composites [30].

These results may be related to the high precision of the spectrophotometric analysis of the specimens, allowing the detection of subtle color differences that are not perceived by the human eye. Spectrophotometers demonstrate a 33% improvement in precision and a more objective match in 93.3% of cases [32, 33]. Furthermore, these devices are not affected by metamerism, which is the ability of two objects to appear to be the same color when illuminated by one light source but to be different when viewed under another [34].

Monochromatic resin composites can cause an optical illusion but not an actual change in the color of the restorative material. Thus, if the restorations were removed from their cavities, they would retain their original color rather than that of the teeth they were placed in. Thus, this optical effect cannot mislead instrumental analysis using a spectrophotometer, but it affects how the clinician sees the restoration. Therefore, the surface characteristics of the restorative material, such as roughness and gloss, are essential for enhancing the metameric behavior of resin composites and improving the optical blending effect with the surrounding dental tissues.

The surface smoothness of resin composites is directly associated with their gloss levels, and materials with higher gloss tend to blend more effectively with adjacent tooth structures compared to those with lower gloss [35]. In a study evaluating different polishing systems and monochromatic resin composites, Omnichroma demonstrated the lowest surface roughness and the highest gloss values, both before and after polishing with all tested systems [35].

Although the CIE L*a*b color model can determine the association between brightness, chromaticity, and hue and enables precise calculation of the colorimetric difference (ΔE), it cannot determine the effects associated with a series of psychophysical and organoleptic effects, including background effects (simultaneous and complementary contrast) and environmental adaptation effects (chromatic and light assimilation) in addition to the structural geometric effects present in specific images [36].

These visual chromatic effects observed in the visual analysis of resin composites can also be explained by the Munker–White illusion. This illusion demonstrates that colors with identical luminance have different perceived brightness when inserted near lighter or darker surfaces. For example, shades of gray appear darker when near white surfaces and lighter when close to black areas [37].

This study, involving an in vitro experiment, has limitations and cannot be immediately extrapolated to clinical situations. The real conditions of the contact of the materials with saliva, variations in mouth temperatures, and effects of brushing and occlusion were not simulated. In the color stability test, the specimens were continuously immersed in coffee, which does not accurately reflect clinical reality but exposes the materials to extreme conditions. Moreover, the lack of simulated mechanical aging (e.g., brushing or wear cycles) can influence the study results. Therefore, clinical studies should be encouraged to confirm the results of this study.

The findings of this study demonstrate that the two monochromatic resin composites exhibited comparable performance to the conventional resin composite in terms of sorption, solubility, color stability, and aesthetic integration with adjacent tooth structures, as assessed by visual analysis. However, the monochromatic resin composites presented lower microhardness values. Despite this limitation, monochromatic resins represent safe and viable alternatives, offering advantages such as simplified clinical protocols, reduced material use, and time savings. In anterior restorations, their use is particularly recommended for less complex cases, where stratification with resins of varying opacities is not required.

## AUTHOR CONTRIBUTIONS

**Conceptualization**: Luís Felipe Espíndola‐Castro. **Formal analysis**: Ericka dos Santos Lopes, Mayara Evelly Duarte de Lima, Emanuel Ewerton Mendonça Vasconcelos. **Investigation**: Ericka dos Santos Lopes, Mayara Evelly Duarte de Lima, Emanuel Ewerton Mendonça Vasconcelos. **Methodology**: Luís Felipe Espíndola‐Castro, Natália Gomes de Oliveira, Gabriela Queiroz de Melo Monteiro. **Writing—original draft**: Ericka dos Santos Lopes, Mayara Evelly Duarte de Lima, Emanuel Ewerton Mendonça Vasconcelos. **Writing—review and editing**: Luís Felipe Espíndola‐Castro, Natália Gomes de Oliveira, Gabriela Queiroz de Melo Monteiro.

## CONFLICT OF INTEREST STATEMENT

The authors declare no competing interests
